# Insulin resistance evaluated by estimated glucose disposal rate and its association with cardiometabolic risk factors among adolescents in Northeast China

**DOI:** 10.3389/fnut.2026.1853266

**Published:** 2026-08-27

**Authors:** Yiwen Wang, Hui Yao, Ling Li, Runyu Du

**Affiliations:** 1 Department of Endocrinology, Shengjing Hospital of China Medical University, Shenyang, China; 2 Department of Endocrinology, Jinghua Hospital, Shenyang, China

**Keywords:** adolescents, cardiometabolic risk, estimated glucose disposal rate, insulin resistance, predictive performance

## Abstract

**Background:**

Insulin resistance (IR) is a key factor in cardiometabolic diseases, but there are few practical ways to measure it in adolescents. The insulin sensitivity’s surrogate measure, the eGDR, has not been well validated in young populations.

**Methods:**

eGDR was determined by the use of waist circumference (WC), HbA1c levels, and hypertension status in this cross-sectional study of Chinese adolescents. Restricted cubic spline models and multivariate logistic regression were utilized to estimate associations between eGDR and cardiometabolic risk factors (CMRFs), such as dyslipidemia, hyperuricemia, and high fasting plasma glucose (FPG). The eGDR’s discriminative performance was compared with traditional IR indices, such as HOMA-IR, TyG, AIP, TyG-BMI, and QUIKI.

**Results:**

After confounders’ adjustment, each SD raise in eGDR was linked with reduced odds of dyslipidemia (OR = 0.781, 95% CI: 0.703–0.866, *p* < 0.001) and hyperuricemia (OR = 0.884, 95% CI: 0.800–0.977, *p* = 0.016). eGDR showed negative correlations with atherogenic lipid subfractions, such as LDL-C, non-HDL-C, and triglycerides (TG), but not with total cholesterol (TC) or FPG. Subgroup analyses showed that the protective effect against dyslipidemia was stronger in participants with BMI in the ≥ 85th percentile, and sex-specific differences in the associations with TG and FPG. eGDR was found to be better than traditional IR indices in discriminating clustered CMRFs. Sex-specific cut-offs for IR were determined: eGDR <8.06 mg/kg/min and <8.46 mg/kg/min for males and females, respectively.

**Conclusion:**

eGDR is a strong, non-invasive cardiometabolic risk indicator in adolescents, especially dyslipidemia and hyperuricemia. It can offer a feasible instrument of early risk stratification in this group.

## Introduction

Cardiovascular diseases (CVD) are the most common mortality cause in the world, and there is a significant amount of evidence that the pathological basis of these diseases is formed in childhood and adolescence and does not manifest itself only in adulthood (1, 2). Insulin resistance (IR) is a major pathogenic agent in various metabolic disorders and is becoming more and more important in the development of CVDs (3–6). China has seen a sharp increase in adolescent overweight and obesity, and sedentary lifestyles over the past decades, with a growing concentration of IR and other metabolic risk factors (7, 8). In turn, the timely diagnosis of IR is of the utmost public health significance to identify adolescents with greater risk of developing CVD.

The hyperinsulinemic-euglycemic clamp, which is commonly deemed the gold standard of insulin sensitivity, is not feasible in large-scale population studies since it is invasive, expensive, and technically challenging (9). Other indices have been created, including the homeostasis model IR assessment (HOMA-IR) and the triglyceride-glucose (TyG) index, but these are based on fasting blood samples and may not capture the entire metabolic picture (10–12).

The eGDR is a new, insulin-independent variable that is determined using waist circumference (WC), glycated hemoglobin (HbA1c), and hypertension status. eGDR has been shown to be a strong predictor of metabolic and cardiovascular outcomes in adults with prediabetes or diabetes (13, 14). Nevertheless, there is limited information on eGDR distribution among adolescent groups and its correlation with cardiometabolic risk factors (CMRFs).

This study studied the relation between IR, as assessed by eGDR, and CMRFs in adolescents from Northeast China. In particular, we described the distribution of eGDR in this population, tested the independent relationship between lower eGDR (reflective of increased IR) and CMRFs, and tested the predictive value of eGDR of cardiometabolic risk.

## Materials and methods

### Study design and data collection

This cross-sectional study utilized data from a survey conducted in urban secondary schools in Liaoyang City, China (December 2010–January 2011). A stratified cluster sampling method was employed, with school grade as the strata and classes as the primary sampling units. Initially, 947 students were recruited. Of these, 936 completed both questionnaires and physical measurements (15). For the present analysis, 894 adolescents were become part of the final analysis after excluding 42 participants with incomplete data. The hospital ethics committee approved the study protocol and informed consent was signed by all participants and their parents or guardians.

### Variables assessed

The variables that were collected and analyzed statistically included the following:

- Demographic and clinical factors: these were sex, age, and family history of Type 2 Diabetes Mellitus (T2DM), which was determined as the presence of T2DM in at least one first- or second-degree family member. Moreover, the time spent on entertainment and time spent on physical activity were measured and evaluated daily and divided into ≤1, ≤2, …, ≤5 h per day.
- Anthropometric measurements: measurements comprised waist to hip ratio (WHR), body mass index (BMI), hip circumference (HC), WC, body weight, and height were then computed based on these parameters.
- Blood pressure indicators: DBP and SBP were measured utilizing standardized procedures.
- Biochemical parameters: blood-based parameters were TC, TG, HDL-C, LDL-C, AST, ALT, HbA1c, fasting insulin (FINS), fasting plasma glucose (FPG), and serum uric acid (SUA).

### Measurement procedures

All physical tests were conducted by professionally trained medical staff in accordance with standard procedures. Anthropometric measurements were taken when the participants were wearing light clothes and barefoot to make sure that the measurements were accurate. Calibrated instruments were used to measure height and body weight, with an accuracy of 0.1 cm and 0.1 kg, respectively. WC was measured at the end of a normal exhalation at the narrowest region between the iliac crest and lower rib margin. HC was assessed at the point of maximum protrusion of the buttocks. A mercury sphygmomanometer was utilized to determine blood pressure after the participants had been seated and allowed to rest at least 10 min. Two readings were made and the mean value was computed to be analyzed later.

Venous blood samples were fasted overnight (≥ 10 h) before collection. At the Liaoyang Diabetes Hospital laboratory, biochemical parameters, such as FPG, TC, TG, HDL-C, AST, ALT, and serum uric The Friedewald formula was utilized to measure LDL-C (16). The central laboratory of Shengjing Hospital, China Medical University measured HbA1c levels using HPLC (D-10 Hemoglobin Testing System, Bio-Rad, USA). The results were standardized in accordance with the Diabetes Control and Complications Trial (DCCT) criteria. Radioimmunoassay method was utilized to note the fasting insulin (FINS) concentrations (Chinese Institute of Atomic Energy, Beijing). IR was assessed by the homeostasis model assessment index (HOMA-IR) which was calculated as: HOMA-IR = [FINS (μU/mL) × FPG (mmol/L)/22.5 (17). The quantitative insulin sensitivity check index (QUICKI) was also used to determine insulin sensitivity, which was defined as: QUICKI = 1/[log FINS (U/mL) + log fasting glucose(mg/dL)] (18). The triglyceride–glucose (TyG) index was determined as: TyG = ln [TG (mg/dL) × glucose (mg/dL)/2] (19). Also, the TyG-BMI index (TyGBMI) was determined by multiplying the TyG index ×BMI (20). The atherogenic index of plasma (AIP) was measured as: AIP = log [TG (mmol/L)/ HDL-C (mmol/L)] (21).

### Definition

The eGDR was estimated by the following equation: eGDR (mg/kg/min) = 21.158 − (0.09 × WC) − (3.407 × hypertension) − (0.551 × HbA1c), where WC is in centimeters, hypertension is a binary variable yes = 1/no = 0, and HbA1c is in % (22). The participants were categorized as normal weight when their BMI was < 85th percentile and overweight or obese when their BMI was at ≥ 85th percentile. Hypertension was considered to be a SBP of ≥ 130 mmHg and/or a DBP of ≥85 mmHg (23).

The cardiometabolic risk assessment is well-known among pediatric populations and has been associated with premature changes in carotid artery structure and functioning (24). The CMRFs in the current study were high levels of fasting glucose, dyslipidemia, and hyperuricemia. Based on the criteria set by the IDF on pediatric metabolic syndrome, the following definitions were used: hypertriglyceridemia was defined as triglyceride levels ≥ 1.70 mmol/L; lower HDL-C was described as <1.03 mmol/L in participants aged 10–15 years, and in individuals of age ≥ 16 years as < 1.03 mmol/L in males and < 1.29 mmol/L in females; high fasting glucose was considered as FPG ≥ 5.6 mmol/L (23). Hypercholesterolemia was considered as TC ≥ 5.2 mmol/L and high levels of LDL-C ≥ 3.4 mmol/L, according to the levels suggested by the American Academy of Pediatrics (25), since the IDF does not provide cut-off values of these parameters in children. Dyslipidemia was considered to have at least one of the following abnormalities: hypertriglyceridemia, hypercholesterolemia, low HDL-C, or high LDL-C. Hyperuricemia was defined as a SUA level ≥ 357 μmol/L, which is in line with commonly used thresholds in similar adolescent studies (26).

Without universally agreed thresholds of IR in adolescents, eGDR cut-off values were determined through a percentile-based approach, which is in line with the strategies used to determine the surrogate markers of IR (27, 28). In particular, two thresholds were established. The initial threshold (Cutoff A) was set as the 10th percentile of eGDR in adolescents with normal BMI and normoglycemia, stratified by sex (8.07 in males and 8.46 in females). The second cutoff (Cutoff B) was the 25th percentile of eGDR in the total sex-specific sample (8.20 in males and 10.30 in females). The participants whose eGDR values were lower than the respective sex-specific thresholds were considered to have IR.

### Statistical analysis

All statistical tests were performed with SPSS 27 and R 4.5.2. Continuous variables are presented as mean ± standard deviation (SD) or median with interquartile range (IQR), as it is suitable, whereas categorical variables are presented as frequencies and percentages. The partial correlation analysis was conducted to assess the associations between eGDR and traditional measures of IR. Multivariate logistic regression models were used to examine the relationships between eGDR and CMRFs with stepwise adjustment of potential confounders, such as sex, age, and other covariates of interest. Restricted cubic spline functions were utilized to test potential dose response relationships. Subgroup analyses were done by sex and BMI and interaction effects were formally tested. Discriminatory performance was assessed via ROC curve analysis (AUC), with internal validation by 1,000 bootstrap resamples to correct for optimism. Decision curve analysis (DCA) was conducted to assess the clinical utility of each model by quantifying the net benefit across a range of clinically relevant threshold probabilities, balancing true positives against false positives. A sensitivity analysis was performed by excluding participants with overweight (BMI ≥ 85th percentile) to assess the robustness of eGDR’s predictive performance. Two eGDR-based IR thresholds were suggested; the consistency of the two thresholds was assessed by the Kappa statistic, and the relationships between the two thresholds and CMRFs were compared by the logistic regression models to determine the best cutoff value. Moreover, all statistical tests were two tailed and a *p*-value < 0.05 was deemed to be a sign of statistical significance.

## Results

### Patient characteristics

Table 1 displays a summary of the baseline data of 894 adolescents (mean age: 13.08 ± 1.44 years; 52.8% male), stratified by quartiles of eGDR. Across increasing eGDR values quartiles (Q1: 7.41 ± 1.06; Q2: 10.35 ± 0.59; Q3: 11.46 ± 0.19; Q4: 12.24 ± 0.35), a consistent downward trend was observed in WC, HC, WHR, body weight, and BMI. Likewise, systolic and DBP, and biochemical indicators such as ALT, SUA, HbA1c, and FINS declined in a progressive manner across quartiles (all *p* < 0.001). Conversely, the level of HDL-C rose substantially with an increase in eGDR (*p* < 0.001). The T2DM‘s family history was least common among the participants in the highest eGDR quartile (Q4, 46.5%).

**Table 1:** Characteristics of participants stratified by quartiles of estimated glucose disposal rate.

Characteristics	Overall	Quartiles of eGDR				*p* value
		Q1	Q2	Q3	Q4	
*n*	894	223	224	221	226	
eGDR	10.37 ± 1.94	7.41 ± 1.06	10.35 ± 0.59	11.46 ± 0.19	12.24 ± 0.35	<0.001
Sex(man)	472 (52.8%)	150 (67.3%)	128 (57.1%)	102 (46.2%)	92 (40.7%)	<0.001
Age	13.8 ± 1.44	14.08 ± 1.46	14 ± 1.52	13.81 ± 1.38	13.31 ± 1.27	<0.001
WC(cm)	76.79 ± 10.55	82.75 ± 12.21	83.82 ± 8.49	74.42 ± 2.7	66.26 ± 3.78	<0.001
HC(cm)	94.32 ± 8.56	99.09 ± 9.17	98.82 ± 7.07	92.24 ± 5.18	87.18 ± 5.85	<0.001
WHR	0.81 ± 0.07	0.83 ± 0.07	0.85 ± 0.06	0.81 ± 0.05	0.76 ± 0.05	<0.001
Height(cm)	163.54 ± 8.43	167.12 ± 8.99	164.99 ± 8.48	162.05 ± 7.35	160.04 ± 7.01	<0.001
Weight (kg)	58.21 ± 14.15	67.65 ± 17.16	64.12 ± 11.8	53.77 ± 7.82	47.38 ± 6.83	<0.001
BMI (kg/m2)	21.6 ± 4.16	24.05 ± 5.03	23.49 ± 3.62	20.46 ± 2.63	18.43 ± 1.8	<0.001
SBP (mmHg)	117.54 ± 13.91	131.94 ± 11.77	116.05 ± 10.57	111.21 ± 10.83	110.95 ± 10.71	<0.001
DBP (mmHg)	72.91 ± 10.82	81.35 ± 11.17	70.52 ± 9.51	69.56 ± 8.94	70.2 ± 8.85	<0.001
TC (mmol/L)	4.49 ± 0.8	4.5 ± 0.76	4.59 ± 0.79	4.5 ± 0.81	4.38 ± 0.83	0.054
TG (mmol/L)	0.94 (0.67 ~ 1.28)	0.97 (0.67 ~ 1.26)	0.94 (0.68 ~ 1.40)	0.89 (0.63 ~ 1.26)	0.945 (0.68 ~ 1.27)	0.345
HDL-C (mmol/L)	1.06 ± 0.26	1.02 ± 0.26	1.03 ± 0.27	1.07 ± 0.26	1.12 ± 0.26	<0.001
LDL-C (mmol/L)	3.36 ± 0.21	3.38 ± 0.19	3.37 ± 0.19	3.37 ± 0.16	3.33 ± 0.28	0.081
AST(U/L)	17 (15 ~ 19)	17 (15 ~ 20)	16 (14 ~ 19)	17 (14 ~ 19)	16 (15 ~ 18.25)	0.073
ALT(U/L)	11 (8 ~ 13)	12 (9 ~ 16)	11 (8 ~ 14)	10 (8 ~ 12)	10 (8 ~ 12)	<0.001
SUA (mmol/l)	301.5 (248 ~ 367.25)	337 (272 ~ 406)	317 (268 ~ 383.5)	292 (238 ~ 345.5)	269 (229.75 ~ 317.25)	<0.001
HbA1c (%)	5.43 ± 0.26	5.45 ± 0.26	5.46 ± 0.28	5.45 ± 0.27	5.36 ± 0.23	<0.001
FPG(mmol/L)	4.76 ± 0.51	4.77 ± 0.56	4.75 ± 0.49	4.79 ± 0.53	4.73 ± 0.45	0.610
FINS (mU/mL)	18 (13 ~ 24)	20 (15 ~ 29)	19.25 (14.88 ~ 25.13)	18 (13.5 ~ 23)	15 (11 ~ 21)	<0.001
Family history of T2DM	493 (55.1%)	131 (58.7%)	142 (63.4%)	115 (52%)	105 (46.5%)	0.002

AST aspartate aminotransferase, ALT alanine aminotransferase, BMI body mass index, DBP diastolic blood pressure, eGDR estimated glucose disposal rate, FPG fasting plasma glucose, FINS fasting insulin, HbA1c glycosylated hemoglobin A1c, HC hip circumference, HDL high density lipoprotein, LDL‑C low density lipoprotein, SBP systolic blood pressure, TC total cholesterol, TG triglycerides, UA uric acid, WC waist circumference, WHR waist-to-hip ratio.

Moreover, there were considerable age, height, and sex differences between eGDR quartiles (all *p* < 0.001), indicating that pubertal development and sex-related physiological variation might affect insulin sensitivity. To consider these variables, the further analyses used multivariate-adjusted models to assess the independent relationship between eGDR and cardiometabolic risk.

### Association between eGDR and CMRFs

**Figure 1:**
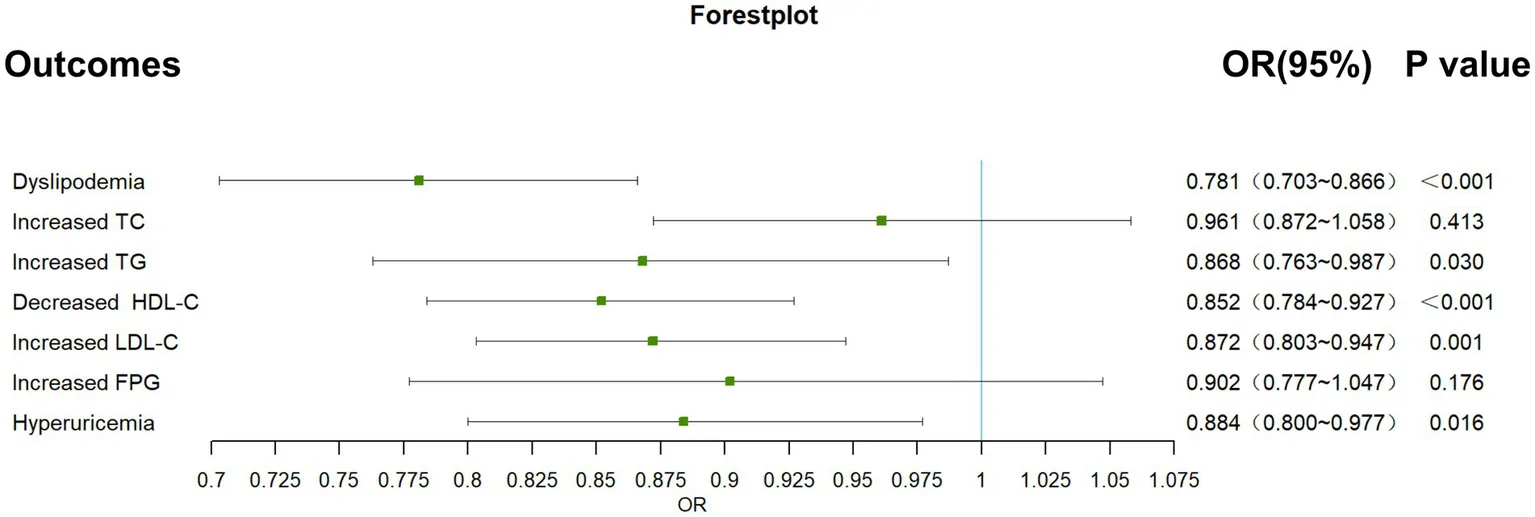
Multivariate-adjusted odds ratios (95% confidence intervals) of estimated glucose disposal rate eGDR for CMRFs in Model 3. Horizontal solid black lines represent 95% CIs; central square markers denote point-estimate OR values; vertical dashed reference line at OR = 1.0 indicates no statistically significant association. All statistical tests two-tailed, *α* = 0.05.The adjusted models adjusted age, sex, height, AST, ALT, daily screen time for entertainment and physical activity duration, family history of T2DM.AST aspartate aminotransferase, ALT alanine aminotransferase, eGDR estimated glucose disposal rate, FPG fasting plasma glucose, HDL-C high‑density lipoprotein cholesterol, LDL‑C low‑density lipoprotein cholesterol, TC total cholesterol, TG triglycerides.

**Figure 2:**
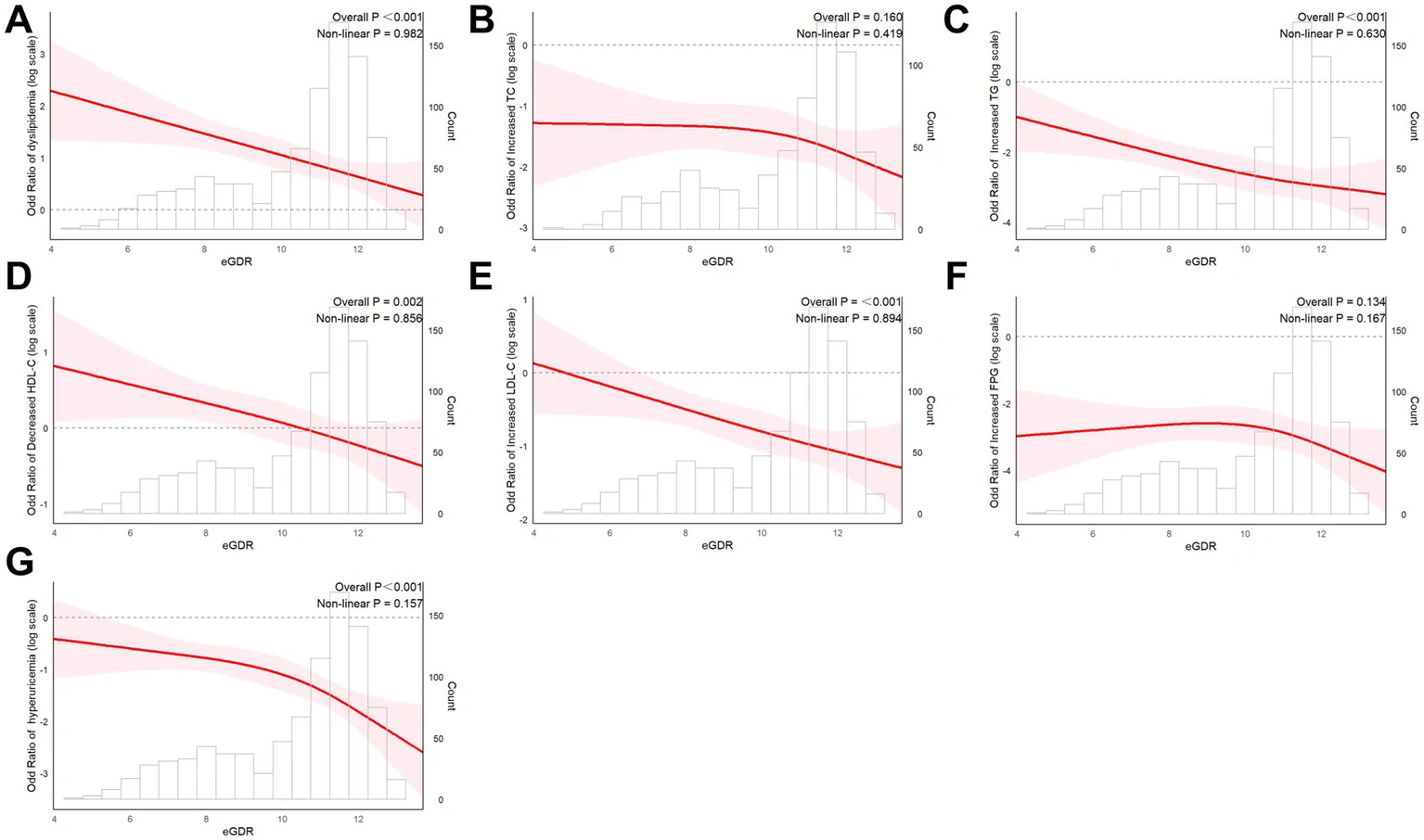
Restricted cubic spline curves for CMRFs according to the eGDR. **(A–E)** dyslipidemia, increased TC and TG, decreased HDL-C, and increased LDL-C; **(F)** elevated FPG; **(G)** hyperuricemia. Odds ratios are indicated by solid lines and 95% CIs by shaded areas. The horizontal dotted line represents the odds ratio of 1.0. The adjusted models adjusted age and sex. Two-tailed statistical testing applied for all trend analyses. AST aspartate aminotransferase, ALT alanine aminotransferase, eGDR estimated glucose disposal rate, FPG fasting plasma glucose, HDL-C high density lipoprotein cholesterol, LDL-C low density lipoprotein cholesterol, TC total cholesterol, TG triglycerides.

Following extensive adjustment of possible confounders (Model 3), eGDR was still significantly correlated with various CMRFs in adolescents (Table 2; Figure 1). Specifically, each one–SD raise in eGDR was linked with a 22% lower likelihood of dyslipidemia (odds ratio [OR] = 0.781, 95% confidence interval [CI]: 0.703–0.866, *p* < 0.001) and a 12% decrease in the odds of hyperuricemia (OR = 0.884, 95% CI: 0.800–0.977, *p* = 0.016). Additional lipid subcomponent analysis showed that increased eGDR scores were always associated with a better lipid profile. For each 1-SD increment in eGDR, the odds of reduced HDL-C reduced by 14.8% (OR = 0.852, 95% CI: 0.784–0.927), while the likelihood of elevated LDL-C and TG declined by 12.8% (OR = 0.872, 95% CI: 0.803–0.947) and 13.2% (OR = 0.868, 95% CI: 0.763–0.987), respectively. These findings were further confirmed by restricted cubic spline analyses, which adjusted by sex and age, showed significant inverse relationships between eGDR and a number of CMRFs (Figure 2). There was a linear and negative correlation between eGDR and dyslipidemia, as well as its separate components (elevated LDL-C, reduced HDL-C, and elevated TG). Conversely, the relationship between eGDR and hyperuricemia was nonlinear. All statistically significant results (P for trend < 0.05; Table 2) were found to have significant dose response relationships. No substantial links were noted between eGDR and elevated TC (OR = 0.961, 95% CI: 0.872–1.058, *p* = 0.413) or elevated FPG (OR = 0.902, 95% CI: 0.777–1.047, *p* = 0.176) (Table 2; Figure 2).

**Table 2:** Multivariate-adjusted odd ratios (95% confidence intervals) of estimated glucose disposal rate for cardiometabolic risk factors.

eGDR	Total N	Model 1		Model 2		Model 3	
		OR (95% CI)	*p* value	OR (95% CI)	*p* value	OR (95% CI)	*p* value
Dyslipodemia							
Per SD increase	894	0.811(0.742 ~ 0.887)	<0.001	0.813(0.741 ~ 0.892)	<0.001	0.781(0.703 ~ 0.866)	<0.001
Quartiles							
Q1	223	Ref		Ref		Ref	
Q2	224	0.848(0.532 ~ 1.354)	0.490	0.832(0.517 ~ 1.338)	0.448	0.745(0.452 ~ 1.227)	0.247
Q3	221	0.565(0.361 ~ 0.884)	0.012	0.544(0.344 ~ 0.862)	0.009	0.488(0.295 ~ 0.806)	0.005
Q4	226	0.427(0.276 ~ 0.661)	<0.001	0.449(0.284 ~ 0.709)	0.001	0.369(0.223 ~ 0.610)	<0.001
P for trend			<0.001		<0.001		<0.001
Increased TC							
Per SD increase	894	0.967(0.889 ~ 1.051)	0.423	0.943(0.864 ~ 1.030)	0.195	0.961(0.872 ~ 1.058)	0.413
Quartiles							
Q1	223	Ref		Ref		Ref	
Q2	224	1.462(0.920 ~ 2.323)	0.108	1.406(0.882 ~ 2.240)	0.152	1.466(0.894 ~ 2.404)	0.130
Q3	221	1.240(0.772 ~ 1.993)	0.374	1.142(0.705 ~ 1.849)	0.590	1.269(0.757 ~ 2.128)	0.366
Q4	226	0.954(0.584 ~ 1.558)	0.850	0.868(0.522 ~ 1.445)	0.586	0.941(0.544 ~ 1.629)	0.828
P for trend			0.675		0.426		0.713
Increased TG							
Per SD increase	894	0.849(0.765 ~ 0.942)	0.002	0.797(0.712 ~ 0.891)	<0.001	0.868(0.763 ~ 0.987)	0.030
Quartiles							
Q1	223	Ref		Ref		Ref	
Q2	224	1.123(0.643 ~ 1.958)	0.684	1.074(0.600 ~ 1.925)	0.810	1.378(0.715 ~ 2.656)	0.338
Q3	221	0.380(0.184 ~ 0.787)	0.009	0.335(0.158 ~ 0.707)	0.004	0.442(0.194 ~ 1.005)	0.051
Q4	226	0.666(0.359 ~ 1.237)	0.198	0.474(0.247 ~ 0.910)	0.025	0.668(0.316 ~ 1.410)	0.289
P for trend			0.029		0.002		0.070
Decreased HDL‑C							
Per SD increase	894	0.838(0.781 ~ 0.898)	<0.001	0.874(0.810 ~ 0.943)	<0.001	0.852(0.784 ~ 0.927)	<0.001
Quartiles							
Q1	223	Ref		Ref		Ref	
Q2	224	0.741(0.510 ~ 1.077)	0.116	0.742(0.499 ~ 1.103)	0.140	0.700(0.462 ~ 1.061)	0.093
Q3	221	0.624(0.429 ~ 0.909)	0.014	0.670(0.449 ~ 0.999)	0.050	0.628(0.409 ~ 0.964)	0.034
Q4	226	0.408(0.279 ~ 0.596)	<0.001	0.538(0.357 ~ 0.809)	0.003	0.474(0.305 ~ 0.737)	0.001
P for trend			<0.001		0.003		0.001
Increased LDL‑C							
Per SD increase	894	0.919(0.857 ~ 0.985)	0.017	0.864(0.802 ~ 0.931)	<0.001	0.872(0.803 ~ 0.947)	0.001
Quartiles							
Q1	223	Ref		Ref		Ref	
Q2	224	0.789(0.538 ~ 1.159)	0.227	0.738(0.498 ~ 1.094)	0.130	0.716(0.473 ~ 1.083)	0.113
Q3	221	0.855(0.583 ~ 1.254)	0.422	0.739(0.498 ~ 1.098)	0.135	0.805(0.527 ~ 1.230)	0.316
Q4	226	0.719(0.489 ~ 1.058)	0.094	0.532(0.353 ~ 0.802)	0.003	0.560(0.360 ~ 0.872)	0.010
P for trend			0.143		0.004		0.022
Increased FPG							
Per SD increase	894	0.936(0.820 ~ 1.068)	0.326	0.900(0.784 ~ 1.032)	0.131	0.902(0.777 ~ 1.047)	0.176
Quartiles							
Q1	223	Ref		Ref		Ref	
Q2	224	0.747(0.354 ~ 1.576)	0.443	0.704(0.327 ~ 1.516)	0.370	0.716(0.473 ~ 1.083)	0.113
Q3	221	0.946(0.465 ~ 1.923)	0.878	0.893(0.430 ~ 1.855)	0.762	0.805(0.527 ~ 1.230)	0.316
Q4	226	0.561(0.251 ~ 1.254)	0.159	0.424(0.184 ~ 0.974)	0.043	0.560(0.360 ~ 0.872)	0.010
P for trend			0.259		0.084		0.095
Hyperuricemia							
Per SD increase	894	0.777(0.714 ~ 0.845)	<0.001	0.810(0.742 ~ 0.885)	<0.001	0.884(0.800 ~ 0.977)	0.016
Quartiles							
Q1	223	Ref		Ref		Ref	
Q2	224	0.878(0.567 ~ 1.360)	0.559	0.948(0.607 ~ 1.478)	0.813	1.080(0.664 ~ 1.755)	0.757
Q3	221	0.480(0.294 ~ 0.784)	0.003	0.559(0.338 ~ 0.924)	0.023	0.781(0.449 ~ 1.359)	0.382
Q4	226	0.217(0.118 ~ 0.398)	<0.001	0.262(0.141 ~ 0.489)	<0.001	0.410(0.210 ~ 0.801)	0.009
P for trend			<0.001		<0.001		0.009

Model 1: unadjusted.

Model 2: adjusted for age, sex.

Model 3: model 2 + further adjusted for height, AST, ALT, daily screen time for entertainment and physical activity duration, family history of T2DM.

AST aspartate aminotransferase, ALT alanine aminotransferase, eGDR estimated glucose disposal rate, FPG fasting plasma glucose, HDL‑C high density lipoprotein, LDL‑C low density lipoprotein, TC total cholesterol, TG triglycerides.

### Subgroup analysis

Sex-stratified analyses identified that the negative relationships between eGDR and the majority of CMRFs were typically similar in males and females (Supplementary Table S1). Nevertheless, there was a high level of sex-based heterogeneity in high levels of triglyceride (TG) (P-interaction = 0.024), with a protective effect evident in males (OR = 0.616, 95% CI: 0.4490.844, *p* < 0.01) but not in females. Another marginally significant interaction was found with higher FPG (P-interaction = 0.050), where higher eGDR was only related to a lower risk in females (OR = 0.541, 95% CI: 0.3280.892, *p* < 0.05). There was no significant sex-modification of the eGDR associations with dyslipidemia, TC, HDL-C, or LDL-C (all P-interaction > 0.05).

Stratified by BMI percentile, the BMI status had a significant effect on the negative correlation between eGDR and dyslipidemia (P-interaction = 0.022). The protective value was significantly higher in participants with BMI ≥ 85th percentile (OR = 0.473, 95% CI: 0.321–0.699, *p* < 0.001) than those with BMI below 85th percentile. The inverse relationships between eGDR and lower HDL-C and higher LDL-C were less significant but more significant and significant only in the higher BMI subgroup. Conversely, BMI status did not have a significant effect in altering the relationships between eGDR and high TC, TG, FPG, or hyperuricemia (Supplementary Table S1).

### Correlation of eGDR with traditional IR indices

Correlation analyses revealed that there were significant correlations between eGDR and various established indices of IR (Supplementary Figure S1). In particular, eGDR was negatively related to HOMA-IR (r = −0.27), TyG (r = −0.077), TyG-BMI (r = −0.49), and the AIP (r = −0.13), which were statistically significant (*p* < 0.05). On the other hand, eGDR had a positive correlation with the quantitative insulin sensitivity index (QUICKI) (r = 0.24, *p* < 0.001). The highest correlation was found between eGDR and TyG-BMI.

### Comparative performance of eGDR in risk prediction

**Figure 3:**
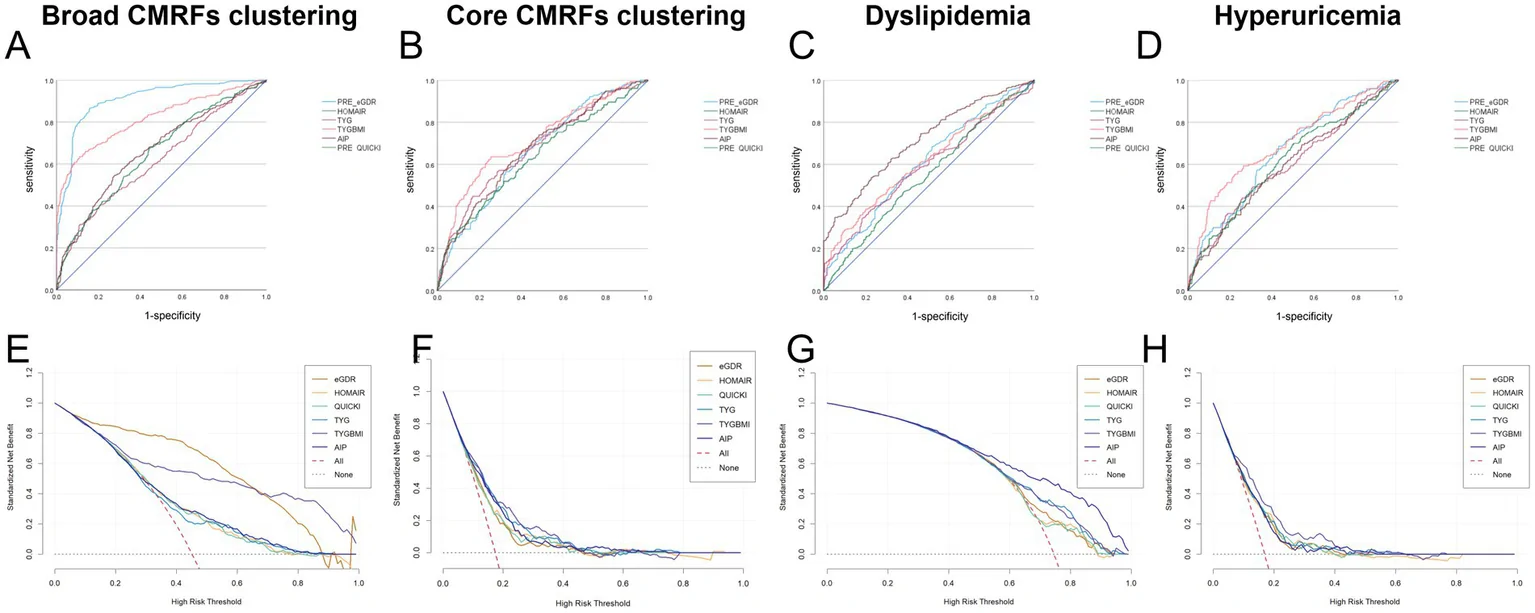
Predictive performance of eGDR versus traditional insulin resistance indices for individual and clustered CMRFs. ROC (left panels: **A–D**) and DCA analysis (right panels: **E–H**) evaluating the discriminative ability of eGDR, HOMA-IR, TyG, TyGBMI, and QUICKI for **(A,E)** broad CMRFs clustering (≥2 of 5 factors: obesity/overweight, hypertension, elevated FPG, dyslipidemia, or hyperuricemia); **(B,F)** core CMRFs clustering (≥2 of 3 core metabolic factors: elevated FPG, dyslipidemia, and hyperuricemia); **(C,G)** dyslipidemia; and **(D,H)** hyperuricemia. DCA curves include models using all indices (“All”, dashed red) and no model (“None”, dotted gray). Standardized net benefit is shown across high-risk thresholds (0–1.0). DeLong’s paired AUC comparison testing performed to quantify statistical differences between eGDR and all traditional IR indices. AIP, atherogenic index of plasma; AUC, area under the ROC curve; DCA, decision curve analysis; eGDR, estimated glucose disposal rate; HOMA-IR, homeostatic model assessment insulin resistance; QUICKI, quantitative insulin sensitivity check index; TyG, triglyceride-glucose index; TyG-BMI, TyG index multiplied by body mass index.

Besides the predetermined CMRFs, two composite clustering outcomes were evaluated. The definition of broad CMRFs clustering was the presence of at least two out of five factors: overweight/obesity, hypertension, high FPG, dyslipidemia, or hyperuricemia. The definition of core CMRFs clustering was the presence of at least three core metabolic elements, including elevated FPG, dyslipidemia, and hyperuricemia. eGDR showed the best predictive ability of Broad CMRFs clustering compared to conventional IR indices, assessed by ROC curves and DCA (Figures 3A,–E; Supplementary Table S2). After adjustment for age and sex and optimism correction, eGDR achieved a bootstrap-validated AUC of 0.892 (95% CI, 0.806–0.972), substantially outperforming HOMA-IR (corrected AUC: 0.523), QUICKI (0.517), TYG (0.515), TyG-BMI (0.539), and AIP (0.532). eGDR was one of the most successful predictors of the clustering of Core CMRFs (Figures 3B,F). Moreover, eGDR was always highly discriminative of individual outcomes, such as dyslipidemia (Figures 3C,G) and hyperuricemia (Figures 3D,H). Sensitivity analyses excluding overweight participants yielded consistent results (Supplementary Table S3; Supplementary Figure S2), confirming the robustness of our findings.

### Comparison of IR definitions and determination of optimal cutoff

We assessed the effect of IR with two different eGDR thresholds (Cutoff A and Cutoff B) on CMRFs using logistic regression analyses (Table 3). Adjusted or unadjusted by age and sex, IR with either cutoff was significantly related to most CMRFs, except high TC and FPG. Even though there was moderate agreement between the two thresholds (*κ* = 0.593, 95% CI: 0.5380648, *p* < 0.001; Table 4), Cutoff A showed better predictive performance on most CMRFs (Table 3). On the basis of these findings, we suggest that Cutoff A should be used to determine IR in adolescents, which is eGDR < 8.06 mg/kg/min in males and eGDR < 8.46 mg/kg/min in females.

**Table 3:** The influence of insulin resistance on cardiometabolic risk factors according to different definitions of insulin resistance.

Items	Groups	cutoffA	*p*	cutoffB	*p*
Dyslipodemia	Crude OR (95% CI)	2.647(1.644 ~ 4.262)	<0.001	1.675(1.203 ~ 2.332)	0.002
	Adjusted OR (95% CI)	2.577(1.586 ~ 4.187)	<0.001	1.538(1.098 ~ 2.154)	0.012
Increased TC	Crude OR (95% CI)	1.044(0.687 ~ 1.586)	0.842	1.083(0.769 ~ 1.525)	0.649
	Adjusted OR (95% CI)	1.099(0.717 ~ 1.684)	0.664	1.065(0.753 ~ 1.506)	0.721
Increased TG	Crude OR (95% CI)	1.754(1.060 ~ 2.902)	0.029	1.393(0.889 ~ 2.182)	0.148
	Adjusted OR (95% CI)	2.219(1.300 ~ 3.789)	0.004	1.720(1.078 ~ 2.745)	0.023
Decreased HDL-C	Crude OR (95% CI)	2.022(1.432 ~ 2.855)	<0.001	1.585(1.203 ~ 2.089)	0.001
	Adjusted OR (95% CI)	1.778(1.231 ~ 2.568)	0.002	1.384(1.032 ~ 1.856)	0.03
Increased LDL‑C	Crude OR (95% CI)	1.463(1.040 ~ 2.058)	0.029	1.195(0.900 ~ 1.587)	0.218
	Adjusted OR (95% CI)	1.737(1.217 ~ 2.478)	0.002	1.312(0.981 ~ 1.756)	0.067
Increased FPG	Crude OR (95% CI)	1.192(0.615 ~ 2.311)	0.602	1.191(0.684 ~ 2.074)	0.537
	Adjusted OR (95% CI)	1.400(0.707 ~ 2.774)	0.334	1.430(0.810 ~ 2.524)	0.218
Hyperuricemia	Crude OR (95% CI)	2.349(1.581 ~ 3.492)	<0.001	1.761(1.234 ~ 2.512)	0.002
	Adjusted OR (95% CI)	2.048(1.361 ~ 3.080)	0.001	1.774(1.232 ~ 2.556)	0.002

Crude OR is the OR before adjusting for sex and age; adjusted OR is the OR after adjusting for sex and age. Clustering of cardiometabolic risk factors: defined as ≥2 cardiometabolic risk factors.

eGDR estimated glucose disposal rate, FPG fasting plasma glucose, HDL‑C high density lipoprotein, LDL‑C low density lipoprotein, TC total cholesterol, TG triglycerides.

**Table 4:** Concordance between the different eGDR cutoff values used in diagnosing insulin resistance.

Groups		IR diagnosed by cutoff B			k (95% CI)	*p*
		−	+	Total		
IR diagnosed by cutoff A	−	577	149	726	0.593 (0.538 ~ 0.648)	<0.001
	+	0	168	168		
	Total	577	317	894		

+ with IR; − without IR. IR, insulin resistance; CI, confidence interval.

## Discussion

IR is a key pathophysiological process involved in the pathogenesis of cardiometabolic diseases (3–6, 29). Obesity and overweight among Chinese adolescents have increased dramatically in recent decades, and IR and clustering of metabolic risk factors have increased in parallel (1, 2, 8). Early detection of IR is thus important in identifying adolescents who are at cardiovascular disease’s raised risk. Traditional IR indices, including the TyG index and HOMA-IR, have their own limitations: HOMA-IR depend on the FINS’s measurement, and the TyG index might not be able to fully reflect the integrated metabolic profile (11, 12). A new measure, the eGDR, based on WC, hypertension status, and HbA1c has been confirmed in adult populations as an effective predictor of cardiovascular risk (13, 14, 30, 31). Nevertheless, the eGDR distribution, its relationships with CMRFs, and its applicability as a screening tool in adolescent groups are not fully described. We have thoroughly investigated the correlations between eGDR and various CMRFs in Chinese adolescents, evaluated its predictive validity compared to traditional IR measures, and sought to determine a clinically useful diagnostic cutoff of IR in this age group.

**Figure 4:**
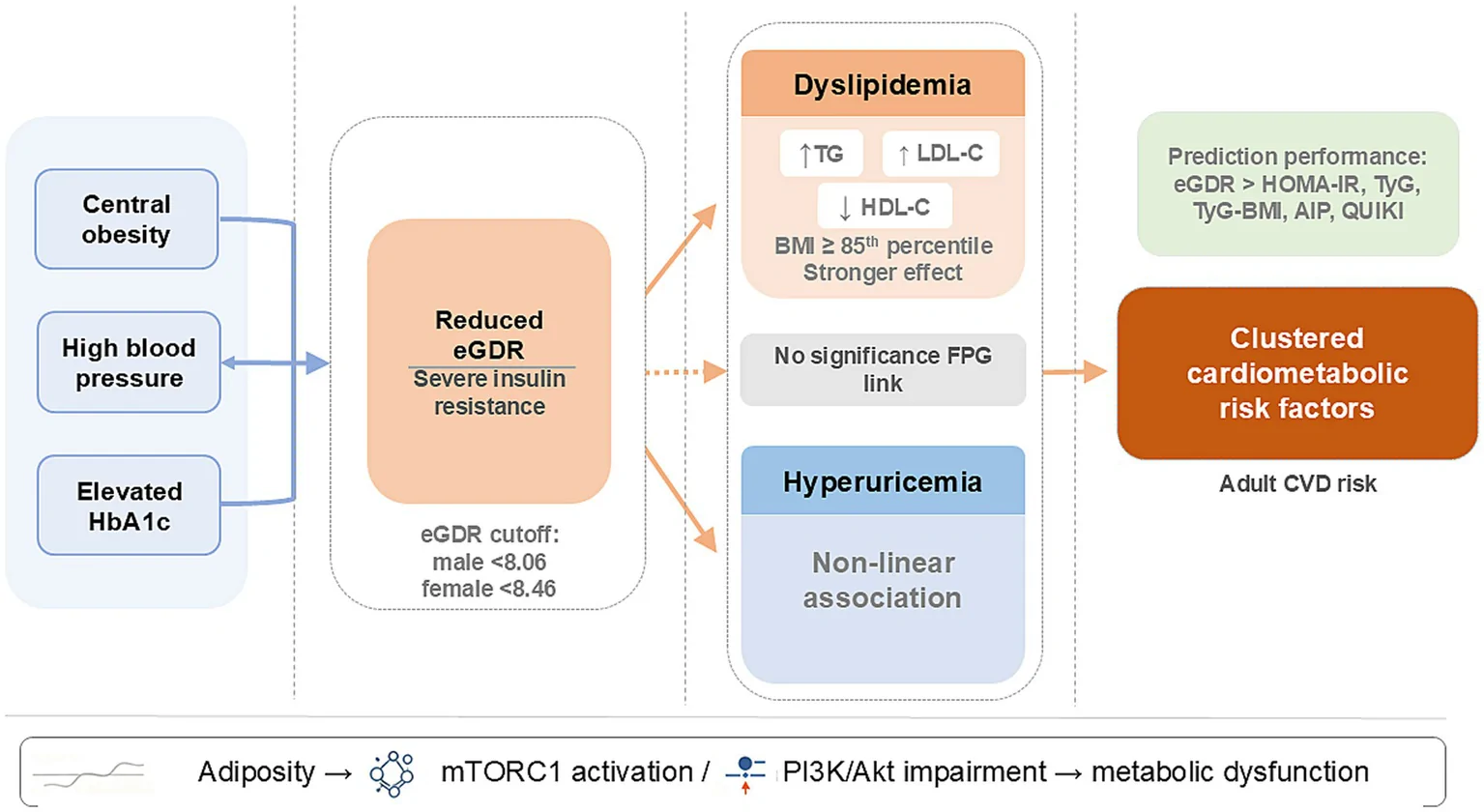
Graphical summary of eGDR and cardiometabolic risk in Chinese adolescents. eGDR, calculated from waist circumference, HbA1c, and hypertension status, is a non-invasive surrogate for insulin resistance. Lower eGDR independently increases risks of dyslipidemia (stronger effect in overweight/obese adolescents with BMI ≥ 85th percentile) and hyperuricemia, but shows no significant association with FPG. eGDR outperforms traditional indices (e.g., HOMA-IR, TyG) in discriminating clustered cardiometabolic risk factors. Sex-specific cut-offs for insulin resistance are <8.06 mg/kg/min for males and <8.46 mg/kg/min for females, supporting early risk stratification. The underlying mechanisms may involve dysregulation of the mTOR and PI3K/Akt signaling pathways. eGDR, estimated glucose disposal rate; TG, triglycerides; LDL-C, low-density lipoprotein cholesterol; HDL-C, high-density lipoprotein cholesterol; FPG, fasting plasma glucose; CVD, cardiovascular disease.

We find that reduced eGDR, which is a marker of increased IR, is independently related to a worse cardiometabolic risk profile in adolescents. This is in line with the findings in adult populations, where eGDR has been repeatedly associated with metabolic syndrome and higher cardiovascular morbidity (32, 33). As illustrated in Figure 4, this study extends the literature by highlighting the early emergence of the relationship between IR and cardiovascular risk. While our data are cross-sectional, they corroborate prospective studies showing that elevated cardiometabolic risk factors during childhood persist and contribute to subclinical cardiovascular damage in adulthood (2, 34, 35), suggesting that the pathophysiological link between IR and CVD begins early in life.

The biological underpinnings linking reduced eGDR to adverse cardiometabolic profiles can be traced to the dysregulation of nutrient-sensing signaling cascades that govern insulin sensitivity, adipose tissue function, and systemic metabolic flexibility (36–38). IR disrupts PI3K/Akt signaling, diminishing glucose uptake in skeletal muscle and adipose tissue, relieving inhibition of hepatic gluconeogenesis, and perturbing lipid metabolism (3, 5, 8). In the setting of IR, adipose tissue dysfunction accelerates lipolysis and raises circulating free fatty acid levels, further perpetuating systemic insulin resistance (8, 29). Recent advances identify mTOR signaling as a core nutrient-sensing pathway that integrates amino acid, energy, and growth factor cues to modulate insulin sensitivity (36–38). The Rab1A–mTORC1–Pdx1 axis governs amino acid sensing within pancreatic *β*-cells and regulates insulin production alongside glucose homeostasis (36). Similar to numerous other signaling cascades (39), mTOR exerts biological effects via sequential phosphorylation events. Specifically, mTORC1-mediated phosphorylation of PDX1 at serine 61 facilitates β-cell proliferation and insulin secretion (38). Excessive caloric intake in adolescents leads to sustained mTOR activation, which drives augmented insulin secretion, hepatic fat accumulation, and progressive insulin resistance (36, 38). Collectively, these mechanistic findings illustrate why eGDR serves as an informative clinical surrogate for insulin resistance. Incorporating waist circumference (a marker of visceral adiposity and adipose inflammation), hypertension (linked to vascular insulin resistance and endothelial dysfunction), and HbA1c (an indicator of glycemic control), eGDR mirrors downstream mTOR dysregulation and compromised whole-body insulin action.

Our results show a clear dose–response relationship: each standard deviation increase in eGDR was linked to a 22% lower risk of dyslipidemia. This highlights how central insulin resistance is to driving the classic atherogenic lipid pattern in adolescents. This effect was especially strong in those with a BMI ≥ 85th percentile. Since insulin resistance is rampant in this group, targeting it might offer a bigger payoff for fixing their lipids. On the other hand, eGDR had no link to total cholesterol. This is expected, as insulin-resistant dyslipidemia is defined by shifts in TG and HDL-C, not necessarily by high TC (3). From a mechanistic perspective, the link between lower eGDR and dyslipidemia can be explained by mTOR-driven fat production in the liver. Overactivation of this nutrient-sensing pathway promotes the synthesis of triglycerides and VLDL particles (39), leading to the classic lipid pattern of IR (high TG and low HDL-C). The stronger association in overweight adolescents fits with this mechanism, as excess calorie intake amplifies this process. Taken together, these findings validate eGDR as a specific tool for catching lipid problems tied to insulin resistance in young people.

It is important to note that eGDR did not have a significant correlation with high FPG in this group of adolescents. This could be an indication of the comparatively low incidence of glucoregulatory impairment among the study population, which implies that, in normoglycemic young people, IR, as measured by eGDR, is mainly expressed through dyslipidemia and hyperuricemia, prior to the development of overt hyperglycemia. This finding is consistent with the idea that IR is a common antecedent, and may be an early predictor of, later glucose metabolism disorders (40). Another plausible explanation for the non-significant association between eGDR and elevated fasting glucose is that eGDR may better reflect postprandial glucose homeostasis instead of overnight fasting glucose. Since OGTT was not performed in the present study, this hypothesis cannot be verified, and we intend to explore this topic in future research. Also, the correlation between eGDR and high TG showed a high level of sexual dimorphism, with a significant negative correlation in males but not in females, indicating that there may be sex-specific processes in lipid metabolism associated with IR.

The negative correlation between eGDR and hyperuricemia in this study, which is equivalent to a 12% decrease in risk per SD increase, is in line with the results of adult populations. Indicatively, NHANES cohort analyses and studies of Chinese adults with type 2 diabetes have also reported such negative associations between eGDR and hyperuricemia risk (41, 42). These results confirm the strength of eGDR as a composite measure of insulin sensitivity and emphasize its applicability to the metabolism of uric acid in various age groups.

It is important to note that eGDR was better at identifying adolescents with clustered CMRFs. Its predictive power, either in a broad definition, including adiposity and hypertension, or in a more restrictive definition, based on dysglycemia, dyslipidemia, and hyperuricemia, was similar or better than that of traditional indices, including HOMA-IR and the TyG index. The DCA findings support that eGDR’s superior clinical utility stems from its integrative nature: it captures central adiposity (WC), blood pressure status, and longer-term glycemic control (HbA1c), three key dimensions of metabolic dysregulation (31). Conversely, traditional IR surrogates reflect only one or two metabolic dimensions, such as hepatic IR (HOMA-IR) or the lipid-glucose interaction (TyG index) (10, 11). This allows eGDR to identify more high-risk adolescents while keeping false positives low, which is particularly valuable in population-based screening settings where cost and intervention resources are limited. These results are consistent with those of adult populations, in which eGDR has been demonstrated to be more effective than other IR surrogates in predicting cardiovascular risk, especially at the initial phases of metabolic dysfunction (43). Together, these outcomes emphasize the usefulness of eGDR as an overall indicator of metabolic health, which can be used as a useful tool to stratify risks in adolescents at an early stage.

This paper suggests sex-specific eGDR cutoffs to determine IR in Chinese adolescents: < 8.06 in males and < 8.46 in females. The IR determined by these thresholds was strongly correlated with most CMRFs, except high TC and FPG. These cutoffs offer a useful, cost-efficient, and insulin-free method of screening in large-scale clinical and community health screening, allowing early detection of at-risk adolescents, even in resource-constrained settings. It is important to note that these sex-specific cut-off values were derived from a single-center cohort in Liaoyang. External validation in independent, multicenter adolescent populations is essential before these thresholds can be adopted for large-scale clinical screening.

There are several of limitations that should be observed. To begin with, this study is cross-sectional, which does not allow causal inference. Second, the sample size can be limited by the use of a single region, which can reduce the generalizability of the results. Third, the lack of a direct comparison to a gold-standard measure of insulin sensitivity, e.g., the euglycemic-hyperinsulinemic clamp, is a methodological limitation. Lastly, the possible impact of pubertal stages of development might not have been taken into consideration.

Future studies ought to concentrate on a few important issues: (1) determining the predictive power of eGDR on hard cardiometabolic outcomes using prospective cohort studies; (2) confirming and, potentially, refining the suggested sex-specific cutoffs in more heterogeneous populations, including rural and multi-ethnic cohorts; (3) using more sophisticated methods, such as imaging of visceral adiposity and omics technologies, to elucidate the underlying pathophysiological mechanisms associated with eGDR; (4) confirming and, potentially, refining the suggested sex-specific cutoffs in more heterogeneous populations, including rural and multi-ethnic cohorts; and (5) assessing the utility of longitudinal eGDR monitoring as a dynamic marker for evaluating the effectiveness of lifestyle or therapeutic interventions.

## Conclusion

Overall, eGDR is a valid and feasible measure that is strongly linked with dyslipidemia, hyperuricemia, and clustered CMRFs among adolescents. It has subcomponent-specific predictive values of lipid abnormalities, with inverse correlations with high TG and LDL-C, and a positive correlation with HDL-C, and no significant correlation with TC. IR as measured by eGDR (cut-offs: < 8.06 in males, < 8.46 in females) is a good predictor of adolescents at high cardiometabolic risk. Being a non-invasive indicator that combines the indicators of central obesity, blood pressure, and glycemic conditions, eGDR has a significant potential in mass screening and early risk stratification of cardiometabolic diseases among adolescents.

## Data Availability

The raw data supporting the conclusions of this article will be made available by the authors, without undue reservation.

## References

[1] Bendor CD Bardugo A Pinhas-Hamiel O Afek A Twig G. Cardiovascular morbidity, diabetes and cancer risk among children and adolescents with severe obesity. Cardiovasc Diabetol. (2020) 19:79. doi: 10.1186/s12933-020-01052-1, 32534575 PMC7293793

[2] Drozdz D Alvarez-Pitti J Wójcik M Borghi C Gabbianelli R Mazur A . Obesity and cardiometabolic risk factors: from childhood to adulthood. Nutrients. (2021) 13:4176. doi: 10.3390/nu13114176, 34836431 PMC8624977

[3] Ormazabal V Nair S Elfeky O Aguayo C Salomon C Zuñiga FA. Association between insulin resistance and the development of cardiovascular disease. Cardiovasc Diabetol. (2018) 17:122. doi: 10.1186/s12933-018-0762-4, 30170598 PMC6119242

[4] Hill MA Yang Y Zhang L Sun Z Jia G Parrish AR . Insulin resistance, cardiovascular stiffening and cardiovascular disease. Metabolism. (2021) 119:154766. doi: 10.1016/j.metabol.2021.154766, 33766485

[5] Accili D Deng Z Liu Q. Insulin resistance in type 2 diabetes mellitus. Nat Rev Endocrinol. (2025) 21:413–26. doi: 10.1038/s41574-025-01114-y, 40247011

[6] Lee SH Park SY Choi CS. Insulin resistance: from mechanisms to therapeutic strategies. Diabetes Metab J. (2022) 46:15–37. doi: 10.4093/dmj.2021.0280, 34965646 PMC8831809

[7] Xin Y Wang H Cai L. Editorial: cardiovascular diseases related to diabetes and obesity - volume IV. Front Endocrinol (Lausanne). (2024) 15:1458742. doi: 10.3389/fendo.2024.1458742, 39092283 PMC11291376

[8] Suren Garg S Kushwaha K Dubey R Gupta J. Association between obesity, inflammation and insulin resistance: insights into signaling pathways and therapeutic interventions. Diabetes Res Clin Pract. (2023) 200:110691. doi: 10.1016/j.diabres.2023.110691, 37150407

[9] Tam CS Xie W Johnson WD Cefalu WT Redman LM Ravussin E. Defining insulin resistance from hyperinsulinemic-euglycemic clamps. Diabetes Care. (2012). 35:1605–10. doi: 10.2337/dc11-2339 PMC3379600 22511259

[10] Tahapary DL Pratisthita LB Fitri NA Marcella C Wafa S Kurniawan F . Challenges in the diagnosis of insulin resistance: focusing on the role of HOMA-IR and Tryglyceride/glucose index. Diabetes Metab Syndr. (2022) 16:102581. doi: 10.1016/j.dsx.2022.102581, 35939943

[11] Tao LC Xu JN Wang TT Hua F Li JJ. Triglyceride-glucose index as a marker in cardiovascular diseases: landscape and limitations. Cardiovasc Diabetol. (2022) 21:68. doi: 10.1186/s12933-022-01511-x, 35524263 PMC9078015

[12] Duan M Zhao X Li S Miao G Bai L Zhang Q . Metabolic score for insulin resistance (METS-IR) predicts all-cause and cardiovascular mortality in the general population: evidence from NHANES 2001-2018. Cardiovasc Diabetol. (2024) 23:243. doi: 10.1186/s12933-024-02334-8, 38987779 PMC11238348

[13] Liao J Wang L Duan L Gong F Zhu H Pan H . Association between estimated glucose disposal rate and cardiovascular diseases in patients with diabetes or prediabetes: a cross-sectional study. Cardiovasc Diabetol. (2025) 24:13. doi: 10.1186/s12933-024-02570-y, 39806389 PMC11730478

[14] Zhang Z Zhao L Lu Y Xiao Y Zhou X. Insulin resistance assessed by estimated glucose disposal rate and risk of incident cardiovascular diseases among individuals without diabetes: findings from a nationwide, population based, prospective cohort study. Cardiovasc Diabetol. (2024) 23:194. doi: 10.1186/s12933-024-02256-5, 38844981 PMC11157942

[15] Du R Li L Li P Wang Y. Impact of insulin resistance on cardiometabolic risk factors and an anthropometry-based predictive nomogram for insulin resistance among adolescents in China. Front Endocrinol (Lausanne). (2022) 13:852395. doi: 10.3389/fendo.2022.852395 35418950 PMC8995502

[16] Friedewald WT Levy RI Fredrickson DS. Estimationof the concentrationof low-density lipoprotein cholesteroiln plasma,without useof the preparative ultracentrifug. Clin Chem. (1972) 18:49. 4337382

[17] Matthews DR Hosker JP Rudenski AS Naylor BA Treacher DF Turner RC. Homeostasis model assessment: insulin resistance and beta-cell function from fasting plasma glucose and insulin concentrations in man. Diabetologia. (1985) 28:412–9. 10.1007/BF00280883 3899825

[18] Katz A Nambi SS Mather K Baron AD Follmann DA Sullivan G . Quantitative insulin sensitivity check index: A Simple, Accurate method for assessing insulin sensitivity in humans *J Clin Endocrinol Metab* (2000) 54:1914–25. 10.1210/jcem.85.7.6661 10902785

[19] Guerrero-Romero F Simental-Mendía LE González-Ortiz M Martínez-Abundis E Ramos-Zavala ḾG Hernández-González SO . The product of triglycerides and glucose, a simple measure of insulin sensitivity. Comparison with the euglycemic-Hyperinsulinemic clamp. J Clin Endocrinol Metabol. (2010) 95:3347–51. doi: 10.1210/jc.2010-0288, 20484475

[20] Dang K Wang X Hu J Zhang Y Cheng L Qi X . The association between triglyceride-glucose index and its combination with obesity indicators and cardiovascular disease: NHANES 2003–2018. Cardiovasc Diabetol. (2024) 23:8. doi: 10.1186/s12933-023-02115-9, 38184598 PMC10771672

[21] Yin B Wu Z Xia Y Xiao S Chen L Li Y. Non-linear association of atherogenic index of plasma with insulin resistance and type 2 diabetes: a cross-sectional study. Cardiovasc Diabetol. (2023) 22:157. doi: 10.1186/s12933-023-01886-5, 37386500 PMC10311747

[22] Williams Katherine V. J R E Dorothy Becker Arslanian Silva Orchard Trevor J. Can clinical factors estimate insulin resistance in type 1 diabetes? Diabetes (2000) 49:626–32. 10.2337/diabetes.49.4.626 10871201

[23] Zimmet P Alberti KG Kaufman F Tajima N Silink M Arslanian S . The metabolic syndrome in children and adolescents– an IDF consensus report. Pediatr Diabetes (2007). 8:299. doi: 10.1111/j.1399-5448.2007.00271.x 17850473

[24] Shah AS Dolan LM Gao Z Kimball TR Urbina EM. Clustering of risk factors: a simple method of detecting cardiovascular disease in youth. Pediatrics. (2011) 127:e312–8. doi: 10.1542/peds.2010-1125, 21242216 PMC3025419

[25] Nutrition., A.A.o.P.a.C.o., American Academy of Pediatrics. Committee on Nutrition Cholesterol in childhood. Pediatrics (1998) 101:141–7. 11345978

[26] Ford ES Li C Cook S Choi HK. Serum concentrations of uric acid and the metabolic syndrome among US children and adolescents. Circulation. (2007) 115:2526–32. doi: 10.1161/CIRCULATIONAHA.106.657627, 17470699

[27] Barja S Arnaiz P Dominguez A Villarroel L Cassis B Castillo O Normal plasma insulin and HOMA values among Chilean children and adolescents. Rev Med Chil (2011). 139:1435–43. 22446648

[28] Song P Yu J Chang X Wang M An L. Prevalence and correlates of metabolic syndrome in Chinese children: the China health and nutrition survey. Nutrients. (2017) 9:79. doi: 10.3390/nu9010079, 28106792 PMC5295123

[29] Gast KB Tjeerdema N Stijnen T Smit JWA Dekkers OM. Insulin resistance and risk of incident cardiovascular events in adults without diabetes: meta-analysis. PLoS One. (2012) 7:e52036. doi: 10.1371/journal.pone.0052036, 23300589 PMC3532497

[30] Wang T Li M Zeng T Hu R Xu Y Xu M . Association between insulin resistance and cardiovascular disease risk varies according to glucose tolerance status: A Nationwide prospective cohort study. Diabetes Care. (2022) 45:1863–72. doi: 10.2337/dc22-0202, 35700159 PMC9346991

[31] Stenlid R el Amrani S Cerenius SY Aydin BK Manell H Mörwald K . Cardiometabolic risk stratification in pediatric obesity: evaluating the clinical utility of fasting insulin and BMI-SDS. Cardiovasc Diabetol. (2025) 24:324. doi: 10.1186/s12933-025-02882-7, 40781672 PMC12335099

[32] Chen X Li A Ma Q. Association of estimated glucose disposal rate with metabolic syndrome prevalence and mortality risks: a population-based study. Cardiovasc Diabetol. (2025) 24:38. doi: 10.1186/s12933-025-02599-7, 39844166 PMC11756087

[33] Zhang J Sun Z Li Y Yang Y Liu W Huang M . Association between the cumulative estimated glucose disposal rate and incident cardiovascular disease in individuals over the age of 50 years and without diabetes: data from two large cohorts in China and the United States. Cardiovasc Diabetol. (2025) 24:51. doi: 10.1186/s12933-025-02575-1, 39891229 PMC11786493

[34] Jacobs DR Jr Woo JG Sinaiko AR Daniels SR Ikonen J Juonala M . Childhood cardiovascular risk factors and adult cardiovascular events. N Engl J Med. (2022) 386:1877–88. doi: 10.1056/NEJMoa2109191 35373933 PMC9563825

[35] Kartiosuo N Raitakari OT Juonala M Viikari JSA Sinaiko AR Venn AJ . Cardiovascular risk factors in childhood and adulthood and cardiovascular disease in middle age. JAMA Netw Open. (2024) 7:e2418148. doi: 10.1001/jamanetworkopen.2024.18148, 38913374 PMC11197443

[36] Fan J Yuan Z Burley SK Libutti SK Zheng XFS. Amino acids control blood glucose levels through mTOR signaling. Eur J Cell Biol. (2022) 101:151240. doi: 10.1016/j.ejcb.2022.151240, 35623230 PMC10035058

[37] Fan J Xu Y. Molecular mechanisms underlying the lifespan and healthspan benefits of dietary restriction across species. Front Genet. (2026) 17:1771707. doi: 10.3389/fgene.2026.1771707, 41695773 PMC12900492

[38] Fan J Zhang X Zhang J Zhao T Burley SK Zheng XFS. PDX1 phosphorylation at S61 by mTORC1 links nutrient signaling to β cell function and metabolic disease. Cell Rep. (2026) 45:116811. doi: 10.1016/j.celrep.2025.116811, 41528843 PMC12949489

[39] Fan J Gao Y Lu Y Wu W Yuan S Wu H . PKAc-directed interaction and phosphorylation of Ptc is required for Hh signaling inhibition in Drosophila. Cell Discov. (2019) 5:44. doi: 10.1038/s41421-019-0112-z, 31636957 PMC6796939

[40] Tabák AG Jokela M Akbaraly TN Brunner EJ Kivimäki M Witte DR. Trajectories of glycaemia, insulin sensitivity, and insulin secretion before diagnosis of type 2 diabetes: an analysis from the Whitehall II study. Lancet. (2009) 373:2215–21. doi: 10.1016/S0140-6736(09)60619-X, 19515410 PMC2726723

[41] Wang Z Liu R Tang F Shen Y. The risk of hyperuricemia assessed by estimated glucose disposal rate. Front Endocrinol. (2025) 16:1567789. doi: 10.3389/fendo.2025.1567789, 40607233 PMC12213406

[42] Tang D Gao P Xie W Wang S Wang X Lu L. Associations between estimated glucose disposal rate and hyperuricemia in patients with type 2 diabetes mellitus: a cross-sectional study. BMC Nephrol. (2025) 26:663. doi: 10.1186/s12882-025-04601-6, 41286669 PMC12645770

[43] Dong B Chen Y Yang X Chen Z Zhang H Gao Y . Estimated glucose disposal rate outperforms other insulin resistance surrogates in predicting incident cardiovascular diseases in cardiovascular-kidney-metabolic syndrome stages 0-3 and the development of a machine learning prediction model: a nationwide prospective cohort study. Cardiovasc Diabetol. (2025) 24:163. doi: 10.1186/s12933-025-02729-1, 40241176 PMC12004813

