# Supplementary material for: Insulin resistance evaluated by estimated glucose disposal rate and its association with cardiometabolic risk factors among adolescents in Northeast China
Source: Front Nutr. 2026 Aug 27;13:1853266. doi: 10.3389/fnut.2026.1853266 (PMC13558436; doi:10.3389/fnut.2026.1853266)
Supplement: Supplementary file 1 [file Table_1.docx]

**Table S1.** Subgroup analysis of the association between estimated glucose disposal rate and cardiometabolic risk factors

| Variables | sex | |  | BMI | |  |
| --- | --- | --- | --- | --- | --- | --- |
|  | Male | Female | P _interaction_ | ＜85% | ≥85% | P interaction |
| Dyslipodemia | 0.613（0.476~0.789）^***^ | 0.657（0.466~0.926）^*^ | 0.442 | 0.888（0.682~1.156） | 0.473（0.321~0.699）^***^ | 0.022 |
| Increased TC | 0.825（0.639~1.065） | 1.018（0.763~1.358） | 0.447 | 1.242（0.908~1.701） | 0.889（0.660~1.196） | 0.098 |
| Increased TG | 0.616（0.449~0.844）^**^ | 1.145（0.697~1.878） | 0.024 | 1.382（0.749~2.552） | 0.869（0.627~1.205） | 0.154 |
| Decreased HDL-C | 0.734（0.596~0.903）^**^ | 0.724（0.555~0.944）^*^ | 0.767 | 0.887（0.696~1.131） | 0.711（0.546~0.927）^*^ | 0.198 |
| Increased LDL-C | 0.770（0.626~0.946）^*^ | 0.777（0.600~1.006）^***^ | 0.526 | 0.867（0.681~1.104） | 0.713（0.551~0.922）^**^ | 0.098 |
| Increased FPG | 1.043（0.721~1.507） | 0.541（0.328~0.892）^*^ | 0.050 | 0.808（0.492~1.327） | 0.875（0.566~1.354） | 0.918 |
| Hyperuricemia | 0.862（0.683~1.087） | 0.636（0.442~0.915）^*^ | 0.241 | 1.079（0.741~1.571） | 0.900（0.682~1.186） | 0.672 |

Adjusted variables included age, height, AST, ALT, daily screen time for entertainment and physical activity duration, family history of T2DM

AST aspartate aminotransferase, ALT alanine aminotransferase, eGDR estimated glucose disposal rate, FPG fasting plasma glucose, HDL-C high‑density lipoprotein cholesterol, LDL‑C low‑density lipoprotein cholesterol, TC total cholesterol, TG triglycerides

*P < 0.05, **P < 0.01, and ***P < 0.001
