# Supplementary material for: Insulin resistance evaluated by estimated glucose disposal rate and its association with cardiometabolic risk factors among adolescents in Northeast China
Source: Front Nutr. 2026 Aug 27;13:1853266. doi: 10.3389/fnut.2026.1853266 (PMC13558436; doi:10.3389/fnut.2026.1853266)
Supplement: Supplementary file 2 [file Table_2.docx]

**Table S2.** Area under the ROC curve (AUC).

|  | Broad CMRFs clustering | | Core CMRFs clustering | | Dyslipidemia | | Hyperuricemia | |
| --- | --- | --- | --- | --- | --- | --- | --- | --- |
|  | AUC | P | AUC | P | AUC | P | AUC | P |
| PRE_eGDR | 0.907 |  | 0.663 |  | 0.608 |  | 0.656 |  |
| HOMAIR | 0.653 | ＜0.001 | 0.635 | 0.347 | 0.548 | 0.027 | 0.616 | 0.166 |
| TYG | 0.619 | ＜0.001 | 0.676 | 0.674 | 0.589 | 0.512 | 0.598 | 0.069 |
| TYGBMI | 0.818 | ＜0.001 | 0.712 | 0.033 | 0.616 | 0.737 | 0.696 | 0.091 |
| AIP | 0.662 | ＜0.001 | 0.670 | 0.813 | 0.715 | ＜0.001 | 0.595 | 0.042 |
| PRE_QUICKI | 0.653 | ＜0.001 | 0.635 | 0.347 | 0.548 | 0.027 | 0.616 | 0.166 |
|  |  |  |  |  |  |  |  |  |

Values are AUC with P values for pairwise comparisons against PRE_eGDR, which were conducted using the DeLong test. Broad CMRFs clustering (≥2 of 5 factors: obesity/overweight, hypertension, elevated FPG, dyslipidemia, or hyperuricemia); Core CMRFs clustering (≥2 of 3 core metabolic factors: elevated FPG, dyslipidemia, and hyperuricemia).

Abbreviations: AIP, atherogenic index of plasma; AUC, area under the curve; CMRFs, cardiometabolic risk factors; eGDR, estimated glucose disposal rate; FPG, fasting plasma glucose; HOMA-IR, homeostatic model assessment of insulin resistance; PRE_eGDR, predicted probability of clustered cardiometabolic risk based on eGDR; QUICKI, quantitative insulin sensitivity check index; TyG, triglyceride-glucose index; TyG-BMI, TyG index multiplied by body mass index.
