# Supplementary material for: Insulin resistance evaluated by estimated glucose disposal rate and its association with cardiometabolic risk factors among adolescents in Northeast China
Source: Front Nutr. 2026 Aug 27;13:1853266. doi: 10.3389/fnut.2026.1853266 (PMC13558436; doi:10.3389/fnut.2026.1853266)
Supplement: Supplementary file 3 [file Table_3.docx]

**Table S3.** Area under the ROC curve (AUC) after excluding overweight participants

|  | Broad CMRFs clustering | | Core CMRFs clustering | | Dyslipidemia | | Hyperuricemia | |
| --- | --- | --- | --- | --- | --- | --- | --- | --- |
|  | AUC | P | AUC | P | AUC | P | AUC | P |
| PRE_eGDR | 0.891 |  | 0.592 |  | 0.578 |  | 0.561 |  |
| HOMAIR | 0.492 | ＜0.001 | 0.564 | 0.594 | 0.523 | 0.131 | 0.509 | 0.334 |
| TYG | 0.485 | ＜0.001 | 0.537 | 0.322 | 0.528 | 0.211 | 0.423 | 0.017 |
| TYGBMI | 0.545 | ＜0.001 | 0.591 | 0.984 | 0.569 | 0.809 | 0.535 | 0.603 |
| AIP | 0.540 | ＜0.001 | 0.550 | 0.409 | 0.664 | 0.021 | 0.443 | 0.023 |
| PRE_QUICKI | 0.492 | ＜0.001 | 0.564 | 0.594 | 0.523 | 0.131 | 0.509 | 0.334 |
|  |  |  |  |  |  |  |  |  |

Values are AUC with P values for pairwise comparisons against PRE_eGDR, which were conducted using the DeLong test. Broad CMRFs clustering (≥2 of 5 factors: obesity/overweight, hypertension, elevated FPG, dyslipidemia, or hyperuricemia); Core CMRFs clustering (≥2 of 3 core metabolic factors: elevated FPG, dyslipidemia, and hyperuricemia).

Abbreviations: AIP, atherogenic index of plasma; AUC, area under the curve; CMRFs, cardiometabolic risk factors; eGDR, estimated glucose disposal rate; FPG, fasting plasma glucose; HOMA-IR, homeostatic model assessment of insulin resistance; PRE_eGDR, predicted probability of clustered cardiometabolic risk based on eGDR; QUICKI, quantitative insulin sensitivity check index; TyG, triglyceride-glucose index; TyG-BMI, TyG index multiplied by body mass index.
